# Modelling computer assisted audit techniques (CAATs) in enhancing the Indonesian public sector

**DOI:** 10.12688/f1000research.121674.1

**Published:** 2022-05-23

**Authors:** Pupung Purnamasari, Noor Afza Amran, Rudy Hartanto

**Affiliations:** 1 Accounting Department, Faculty Economics and Business, Universitas Islam Bandung, Bandung, West Java, 40611, Indonesia; 2 Tunku Puteri Intan Safinaz School of Accountancy, Universiti Utara Malaysia, Sintok Kedah, Kedah Darul Aman, 06010, Malaysia

**Keywords:** Audit support, Effectiveness, Efficiency, Computer-assisted audit techniques (CAATs), Public sector

## Abstract

**Background:** This study aims to examine public sector auditors' tendency to use somputer assisted audit techniques (CAATs) in managing their audit works.

**Methods:** A total of 400 questionnaires were distributed to auditors working in the public sectors in Central Java, West Java, and East Java. From the total, 225 questionnaires were returned and completed.  The Structural Equation Modelling (SEM) and Partial Least Square (PLS) were used to analyze the data.

**Results:** The empirical findings reveal that performance expectation and facilitating conditions have encouraged auditors to use CAATs in their works. Further, there is a positive influence between the intention to use and CAATs audit. This implies that auditors with an intention will be more open to using the CAATs optimally in achieving effective and efficient work. The utilization of CAATs in public services needs to have strong support from the government and positive attitudes from the auditors as the users of the system.

**Conclusion:** This study covers broad areas of Central Java, West Java, and East Java. Further, the findings add to the literature on emerging markets specifically for Indonesian government auditors' intention and appropriateness of using CAATs. The use of CAATs help to provide auditors information on the highest number of auditees involved in corruption.

## Introduction

Many organizations prefer to use information technology to effectively develop and enhance their business. ^1^ The use of information technology has also been an effective tool in increasing the quality of public services. ^2^ In addition to its positive impact, such effective information technology in business, public, and private sectors can bring risks and new vulnerabilities in a fully automatic setting. With the use of a novel auditing method, such risk and vulnerability can be recognized, mitigated, and controlled. ^3^ As a result, the development of the audit profession must keep abreast with the evolution of its surroundings. ^4^ A new audit technique includes the use of computer-assisted tools and technologies. ^5^ Examining auditors’ acceptance of the use of computer-assisted audit tools (CAATs) is significant because researchers and practitioners believe that using CAATs will improve an audit’s efficiency, effectiveness, and functionality. ^5^^–^^8^

CAATs are used by auditors in various nations, both internally and externally, in public and commercial sectors. CAATs have been used in the private sector to keep up with technological advancements in large corporations. ^5^^,^^9^^,^^10^ The adoption of CAATs by public-sector auditors has coincided with the shift to information technology-based public-sector auditing, or what is known as e-government. The use of CAATs by auditors in public sectors has been in parallel with the change of information technology-based public service. E-Government refers to any government or institution’s use of information technology to modify how they communicate and interact with citizens, businesses, and other government entities. ^11^

CAATs is a model in inspecting as a course of study/discipline. In contrast to the old-style auditing in medium-sized practices, auditing in the information systems is yet in its primary stages. ^12^ Nevertheless, the International Federation of Accountants (IFAC) has established the International Standards on Auditing (ISA). ^13^ In most scenarios, the auditors are organized to assure the appropriateness and effectiveness of the inner controls executed in the information systems. Moreover, it can be concluded that the speedy variations in the technology have familiarized novel approaches on the required ways the assessment should conduct. Consequently, the former-style audit functions have been challenged by the information system’s usage. ^13^

Moreover, the constantly developing technology has made implementing the audit functions through typical auditing techniques complicated for the assessors. ^14^ The audit’s focus must be shifted from manual recognition to technology-based prevention. ^15^ Many well-established devices have been designed to help the auditors in accomplishing the auditing purposes. Such as the CAATs were proposed for assisting the auditors with the auditing tasks on screening accountancy data. The auditors employed Generalized Audit Software (GAS) for scrutinizing and auditing live or mined info from an extensive stream of applications. ^16^ The software further involves multiple tools allowing the data mining from a customer’s system and examining that data; arithmetical examination, and audit practiced organizations. ^16^^,^^17^

The situation is definite that to empower auditors to subject best accounting data and make simultaneous decisions, have and depend on quality information and exist on the web. Analysts in this area ensure that continuous information is fundamental for precision results in the inspecting space. At the point when reviewers acquire electronic information, they can deal with it all the more deftly. ^16^ This information is without a doubt open; adaptable; it is saved, consolidated, and coordinated in a way better compared to paper-based bookkeeping information. Journalists in the inspecting area proposed that information advancements drive firms to do their exchanges electronically. They will actually want to give their budget reports electronically and online through the ongoing framework. At the latest occasions and under the umbrella of the constant bookkeeping real-time auditing (RTA) frameworks, monetary information might be handled electronically just as having review proof accessible in an electric structure. This necessary organizations to supplant their term paper and customary archives with automatic ones, including orders for buying; solicitations; and checks. ^18^ Evaluating Practice Regulation 1009 “PC Assisted Audit Techniques” (CAATs) is created dependent on the International Auditing Practice Regulation “PC Assisted Audit Techniques” was endorsed by the International Federation of Accountants (IFAC) in the 2001 version. ^19^ Evaluating companies and inspecting experts have presented various CAATs. These methods have been better-quality to help evaluators, completing their inspecting drives relying upon mechanized bookkeeping data. The idea of modernized bookkeeping data frameworks has discovered its direction into the universe of bookkeeping accordingly. The most critical and broadly utilized CAATs in electronic evaluation is Generalized Audit Software, truncated as (GAS) (Singleton and CISA, 2006). Inspectors use GAS to investigate and review live or separate information from an extensive scope of utilizations. ^16^

The CAATs systems offer multiple possibilities. It has employed the computer in place of an auditing tool to increase the auditing method’s usefulness and productivity. ^20^ The purpose of the following research is to observe the effects of computer-assisted audit tools on the establishment of auditing approaches by the auditor’s performance expectations. The study gives an overview of the CAATs technology usage and implications. The possible reason that the auditors started considering CAATs might be the determining resource issue and the distinctive operator perceptions. Previous information system studies signify that even after the availability of enough resources for purchasing IT, the users might not use the new one. ^21^ Information system studies have established multiple samples for predicting the auditor’s IT consideration.

The utilization of CAATs by inward reviewers is not new; however it has developed after some time as the expansion of data innovation utilization has created in organizations. ^22^ The unavoidable idea of data innovation, the ideal monetary and functional adaptability of present-day figuring innovation, and the internationally open and cutthroat a publicise influences that drive the pace of innovative development are together making a period of significant change in the commercial center for review robotization. ^23^^–^^25^ While reviewers have been somewhat effective in utilizing existing advances to robotize components of their capacities, the organizations they work with are likewise going through critical change themselves. ^26^ Numerous associations have picked to use refined data advances for fostering their business cycle support, just as working on their data handling exercises. ^22^ This expands the requirement for CAATs in such organizations to permit examiners to keep on having the option to play out their audit and observing assignments successfully, just as to assume essential parts during the time spent developing in these organizations all the more by and large.

A large number of these commercial environment alterations are having a solid effect on the advancement of the inner and outer review and affirmation trade. ^27^^,^^28^ Inspectors’ strategies must keep up with the auditee business’s administration, and detailing variations to guarantee the viability and efficiencies of the review capacity can be kept up. More extensive utilization of CAATs has been generally promoted as a significant reaction to these changes. ^16^^,^^29^ A large number of scholarly investigations have been led that expression to aid more vast conception of the problems of CAATs reception – less still that especially center around their reception by interior evaluators. ^18^^,^^30^ Accordingly, there is the circumstance for additional investigations to be embraced to give a superior the inspirational comprehension and requirements on the utilization of CAATs in inner review offices. This exploration endeavors to deliver further knowledge on these issuers by and large.

CAATs are thought to minimize audit expenses, improve audit characteristics and efficiency, support suitable reports of the auditing, and increase audit efficiency for auditors and firms that use them. ^6^^,^^31^^,^^32^ Despite its merits, auditors do not use it regularly or constantly for either external auditors ^5^^,^^16^^,^^33^^,^^34^ or public sector auditors. ^35^ This is owing to the fact that there are only a few advantages of adopting CAATs for a small number of clients. ^33^

The International Organization of Supreme Audit Institutions (INTOSAI) began to produce a guidance book on using audit information technology in 2014 for the government audit board in the context of the use of CAATs in public or governmental sectors. After that, INTOSAI, of which Indonesia is a member, has developed the CAATs. As the government audit board in Indonesia is known as the Indonesian Audit Board (BPK), has established four types of CAATs systems for the audit process, which are: Audit Management System (SMP), Audit Application System (SIAP), E-audit, and Follow-up Monitoring Information System (SIPTL).

An established system’s availability has encouraged Indonesian government auditors to adopt the newest or most recent audit technique known as CAATs. However, several studies have found the inclination in the acceptance of CAATs, ^12^^,^^36^^,^^37^ as well as a low level of the use of CAATs by auditors. ^33^^,^^38^^,^^39^ In light of these circumstances, the objective of this study is to examine how effective CAATs are in assisting BPK auditors in doing their audit works in Indonesian public services. This study employed a model for predicting information technology adoption, namely, the use of technology acceptance and use of technology (UTAUT). ^40^ A novel technology, Unified Theory of Acceptance and Use of Technology (UTAUT) combines numerous formerly accepted samples to measure the acceptance probability of novel technologies. Acknowledging the factors responsible for the acceptance enables the examiners and audit firm administration to perceptively develop involvements aimed at the auditors who are unlikely to accept and employ novel systems. As per the UTAUT, four aspects effects user acceptance: (1) the user’s expectancy of a system to enhance their performance, (2) the efforts required to function the new structure, (3) the feedback of the structure by the worker’s trusted sources (e.g., societal impact), and lastly (4) the user’s expectancy about the presence of a technical and organizational set-up, supporting the system’s use (e.g., service settings).

UTAUT combines many formerly established models for determining the probability of success when implementing fresh technologies. The adoption trigger allows researchers and audit firm management to plan proactive interventions (such as training and marketing) for auditors who are less inclined to embrace and use a new system. ^40^ The CAATs involve technologies like electronic audit-working papers, applications of the dataset, as well as business intelligence audit software. ^5^^,^^29^^,^^41^ Despite the CAATs significance in decreasing the cost of an audit, enhancing the value and efficiency of audit, ^42^^,^^43^ and their broad use in advanced states, ^43^ the CAATs is yet not used commonly in the evolving countries. ^44^^,^^45^ The reason for such limited acceptance of CAATs is the audits that are conducted unproductively, or even worst, requiring quality. An investigation on the limitation of CAATs acceptance in such framework is required. Even though the study on CAATs and further technology’s acceptance have concentrated on the organizational aspects (i.e., size of the firms, IT capability of employees, and top administration commitment) and atmospheric aspects (i.e., competitive burdens), however, such elements exclude crucial atmospheric variables which are exclusive for CAATs acceptance. ^46^^,^^47^ The CAATs acceptance is diverse compared to the other technological acceptance typically seen in specific businesses such as industrial and marketing. Three aspects are exclusive for the outer auditing atmosphere: customer’s AISb environment’s complication, the competence pressure from the other auditing firms for CAATs acceptance, and lastly, the degree of support from the qualified accounting organizations for CAATs acceptance. Primarily, customers having the complicated AIS are more expected to appoint bigger audit firms with the ability and enough investments in IT resources and knowledge. ^48^ The competitive pressure for CAATs acceptance is even complicated as compared to other industries. Audit firms confront increased pressure for meeting their financial plans, the need to collect enough verifications, and offer a competitive audit within their assigned budget and time. ^48^ Finally, Professional Accounting Bodies (PABs) control the audit industry, which performs a key part to impact audit firms for the acceptance of CAATs. As per the previous studies on technological acceptance, the central purpose of the following study is to analyze within an evolving state context if the organizational and atmospheric aspects exclusive to CAATs have any impact on the CAATs acceptance across audit firms.

## Literature review

The use of information technology (IT) has gained much attention in many organizations in recent years. This enlightened that computers and information systems become a necessity for business to complete their daily tasks. With the use of technology, businesses have evolved to collect and disclose their financial data. This helps organizations spend less time in paper-based presentation of their financial data and spend more time on their firms’ performance. ^49^ Consequently, many businesses are now attentive towards e-business (electronic business) and investing in complex IT software. ^50^ Various researches emphasize explicitly PC-based audit support networks. For instance, Mansour ^50^ recorded the effect of innovation on review arranging in five huge audit firms and stated that innovation could be utilized to give customers direct internal controls that help the inspector recognize the defects in the client’s frameworks. He additionally found that innovation is helpful for breaking down the client’s business measures, decide and survey the degree of controls, and prescribe tests that should be performed. Moreover, innovation ensures consistency with review principles and other review-related guidelines. Bierstaker, Burnaby ^51^ portrayed the utilization of innovation on the review cycle by talking to IT experts from four of the five biggest US bookkeeping firms. Bierstaker, Burnaby ^51^ studies were graphic in nature and zeroed in on a particular audit application from one audit firm; consequently, the two examinations are not generalizable to the external auditor’s actual utilization of innovation. The examined auditors’ view of the significance of CAATs and the degree of CAATs use. They additionally analyzed how the apparent significance and degree of IT use vary by firm size and over the long run. They discovered firm size had been displayed to be critical in deciding CAATs utilization. Auditors from Big 4 firms (the leading players in the accountancy industry, with their services spanning advisory, audit and assurance, tax, risk consulting and management consulting, and capital and transaction management) were almost certain that more modest firms to utilize IT review applications. The investigation likewise discovered that the hole between IT use for Big 4 and Non-Big 4 firms has been shutting contrasted with the 2004 information. The Non-Big 4 firms’ CAATs utilization nearly looked like that of the Big 4 firms. ^43^ Earlier CAATs learns at the singular level utilized the Technology Acceptance Model (TAM) and Unified Theory of Acceptance, furthermore, Use of Technology (UTAUT) hypothesis. These hypotheses center around innovation trademark factors like performance expectancy, social impact, working with conditions, and perceived usefulness. ^28^^,^^52^

Studies have analyzed the aspects affecting the acceptance of CAATs. The previous studies on CAATs discover its acceptance from the specific level of auditors but not among the audit firms from the organizational perspective. The adoption of CAATs should initiate from an organization’s choice to obtain the technology, invest fundamentally, and offer the services for acceptance for individual auditor’s use. Hence, the firm’s degree of audit technology investment might adequately denote audit technology adoption instead of the individual auditor’s viewpoints. ^5^ Just few studies have examined CAATs’ acceptance from the organizational point. Ismail and Abidin ^53^ described the outcomes of the descriptive analysis regarding IT knowledge and the significance of audit technology across Malaysian audit firms. Ramen, Jugurnath ^1^ postulated several aspects influencing the CAATs acceptance and analyzed the association and the major differences among the individual and organizational-standard aspects. A qualitative study was conducted to discuss the elements capable of influencing the CAATs and recognized Prescribed Accountancy Body (PAB) support as one of them. The auditor’s identification of the CAATs significance and the degree of CAATs usage was investigated by. ^43^ It was also inspected that in which ways the recognized cruciality and scope of IT usage differs from the size of the firm and time. The observation revealed that it was the firm’s size that is vital in considering CAATs use. The auditors from the Big 4 firms were most likely to employ the IT audit applications. The research also revealed that the gap between IT usage for the Big 4 and Non-Big 4 firms was filling, as per the former information. The earlier individual-level CAATs studies have utilized TAM and the UTAUT model. The theories concentrate on the technology features, such as performance expectations, societal impacts, service-providing situations, and recognized practicality. ^53^^,^^54^ The majority of the studies analyzed if the variables in the present UTAUT theoretical context might be implemented to CAATs acceptance in the exterior assessment settings. ^5^^,^^55^

### UTAUT model

UTAUT is a basic theory that can be used to determine e-audit or electornic audit acceptance by individual auditors, which can impact an organization’s technological acceptance. Venkatesh, Morris ^40^ initially proposed UTAUT, which contains four essential predictors of intention and usage: performance expectancy, effort expectancy, social influence, and facilitating condition. Furthermore, UTAUT is a theory that takes genders, age, and experiences into account. ^40^ The use of information technology in the form of an application would be more readily accepted by its users, even if the UTAUT model assumes that auditors, particularly those who have received training on the use of information technology, will be more likely to use Computer Assisted Audit Techniques (CAATs) if it is simple to use. ^34^^,^^56^

### E-Audit

*E-Audit* is a technology-based information system that allows auditors to commit to their auditing tasks easily. The usage of *E-Audit* or CAATs has been widely used in the private sector in several nations. CAATs are used to combat and detect fraudulent activity and dangers. ^57^ The e-Audit system model can be applied through a specific electronic audit program and information technology tools. According to Liang, Lin, ^58^ the Internet’s massive rise has prompted the development of various modern information technologies, including object-oriented middlewares, Internet security technology, and smart agents. Computer-Assisted Audit Techniques (CAAT) can be employed more successfully with information technology that emerges from a new approach to Electronic Data Processing (EDP) audits. Shaikh ^59^ proposed using CAAT, which is built on an electronic audit framework that incorporates most of the present capabilities of audit software but can be designed and distributed independently of the EDP auditors. CAAT, according to Zhao, Yen, ^6^ is required to conduct ongoing audits in the electronic audit process. Because the auditor is a significant actor who has access to the party’s database being audited, the auditor’s capacity and competency are required.

### Implementation of CAATs at BPK

The usage of e-audit was included in BPK’s strategic planning (Renstra) for 2011 to 2014 as the first step in establishing CAATs. The four major components of the e-audit implementation are BPK’s internal information system (e-BPK), BPK’s data warehouse (BPK data warehouse), BPK’s center of access and analysis (BPK Command Centre), and BPK’s internal information system (e-BPK). For BPK, e-audit can be utilized as an early warning system in the event of fraud in public finance management, which will eventually inspire accountability in managing state finances. ^60^ Auditors should keep pace electronically with their customers. Customers need help in introducing and overhauling their endeavour-wide registering stages. Respondents show that it takes a few years for an organization to totally move their old programming to big business-wide figuring stages regularly. In these circumstances, examiners should work with their customers to guarantee that all controls and execution measures expected to assess every business interaction are set up. ^51^ As examining programming is coordinated into the review interaction, evaluators will have more opportunities to resolve their customers’ perplexing issues in the worldwide commercial center. Customer, the board, should foster techniques that fuse the organization’s targets and objectives into execution estimates that can rapidly feature when an interaction is not performing up to norms. Evaluators can utilize CAATs in creating quantifiable targets and execution markers that enterprise wide processing frameworks can serve to electronically screen.

### Performance expectancy

How much somebody accepts that taking on a specific apparatus would assist him with accomplishing more prominent importance is referred to as Performance expectancy. ^40^^,^^61^ Execution hope insinuates the degrees to which an individual acknowledges that using the device can accomplish work execution gains. ^40^ According to Jakšić ^62^ and Saygili, ^63^ commentators who acknowledge that the gathering of CAATs might overhaul their audit convenience and the idea of survey work ought to have uplifting objectives to take on the advancement. They moreover found that the audit specialists’ dynamic communication was updated by electronic demonstration of accounting information. ^64^ Plus, analysts’ conviction that using CAATs will chip away at coordinating audit preliminaries of controls and impressive testing will likely have significant standards to accept CAATs as shown by Bedard, Jackson ^65^ and Loraas and Wolfe. ^66^ Withstanding these benefits, the researcher acknowledges that analysts’ perspective on the support and productivity they desire to gain from using CAATs in their assessing space will quite affect the assumption to embrace and use them. Execution anticipation alludes to the degree to which an individual accepts that utilizing the device can support accomplish gains in work execution. ^40^ Banker, Chang ^64^ found that utilizing CAATs in large review firms diminishes the review time required for working paper arrangement. They likewise tracked down that the review experts’ dynamic interaction was upgraded by the electronic show of bookkeeping data. ^64^ Besides, examiners’ conviction that utilizing CAATs will work on the productivity of leading review trial of controls and meaningful testing is probably going to have high aims to embrace CAATs as indicated by ^65^ and Loraas and Wolfe. ^66^ Withstanding these advantages, the specialist accepts that examiners’ impression of the convenience and usefulness they hope to acquire from utilizing CAATs in their reviewing area will certainly impact the expectation to take on and use them. In summation, the past research demonstrates that the effect of IT on the review cycle has been huge in numerous ways. This adds to the improvement of the audit interaction. ^31^^,^^41^

For example, an auditor might feel that utilizing CAATs will help him fulfill his audit time budget because it minimizes the number of hours spent on substantive testing and controlling, which will lead to an increase in audit time efficiency. ^5^ Many studies show that the use of General Audit Software (GAS) by internal auditors, ^29^^,^^67^ external auditors, ^9^^,^^50^ and legal auditors ^68^ is influenced by performance expectancy. Based on the following studies, it is hypothesized that:

*H _1_=Performance expectancy influences the intention to use CAATs.*

### Effort expectancy

Effort expectancy refers to ‘the ease level related to the use of tools’. ^40^ Janvrin, Bierstaker ^5^ illustrated how comfortable those auditors who have received training are using CAATs. Hoque, Saif ^56^ also found that users can accept an application if it is easy to use. Research by ^68^ showed that effort expectancy influences the use of CAATs by legal auditors. Venkatesh, Morris ^40^ contend that effort expectancy is relied upon to be more notable in the beginning phases of another conduct when interaction issues address obstacles to survive and later become superseded by instrumentality concerns. Payne and Curtis ^34^ note that since inspectors may settle on the choice to take on innovation and be answerable for carrying out the innovation, the work associated with innovation reception might be more notable to evaluators than to other IT experts. Hence, Payne and Curtis ^34^ contend that work hope will be related to a social goal. Furthermore, a study by ^67^ revealed that the expectation of work also influences the use of CAATs by internal auditors. The second hypothesis presumed that:

*H _2_=The effort expectancy influences the intention of using CAAT’s.*

### Social influence

Social influence can be defined as ‘the extent to which someone perceives the importance of others to use the new tool. ^40^ In the context of an audit, this study expects the extent to which auditors perceive their direct managers’ support the use of CAATs can influence whether they adopt CAATs or not. ^5^ The earlier examination has demonstrated that social influence affects the client’s aim to acknowledge and use an innovation. ^69^^,^^70^ In a review setting, we expect that the more noteworthy examiners see that their immediate administrators support CAATs utilization, the more certain inspectors are to take on CAATs. Loraas and Wolfe ^66^ find that help from companions and support from managers decidedly impacts social aim. Hence, we foresee that social impact will positively influence CAATs use. Several studies have reported that the use of General Auditing Software (GAS) by external auditors is influenced by social influence. ^1^ Based on past studies, this study hypothesized that:

*H _3_=Social influence affects the intention of using CAATs.*

### Facilitating condition

Facilitating condition is defined as ‘the extent to which people believe that the existing organizations have infrastructure and technique to support the use of tools’. ^40^ In the context of the Indonesian public sector of auditing, infrastructures may involve *Kantor Akuntan Publik* (KAP) providing services based on human resources of CAATs and the support of computers for staff such as special instructions, support center, hotline, and the use of guidance. ^71^ Many studies have demonstrated that the use of General Auditing Software by internal auditors, ^29^ external auditors, ^1^^,^^9^^,^^50^ and legal auditors ^68^ are influenced by the facilitating condition. Therefore, based on the above arguments, this study hypothesized that:

*H _4_=Facilitating condition influences the appropriate use of CAATs.*

*The intention of usage*

Individual motivation to do an activity is captured by intention. ^72^ Despite the fact that various circumstances influence how auditors want to utilize auditing support systems, the theory of planned behavior suggests that auditors who want to use the system properly will be more likely to do so. ^72^ Ajzen ^72^ showed that business intelligence (BI) is the most general indicator of conduct and is proposed to be a predecessor of genuine use. UTAUT’s goal to embrace and utilize CAATs is the reliant variable in the exploration model and is an element of execution anticipation, exertion, hope, social impact, and working with conditions. ^21^^,^^73^ This build puts the first ‘goal to utilize’ develop in the CAATs’ setting. Furthermore, ^74^ found that purpose and external factors influence the optimal usage of supporting auditing systems. Thus, the following hypothesis presumed that:

*H _5_=Intention of using CAATs influences the use of appropriate use of CAATs.*

*Moderation model of CAATs acceptance*

Additionally, Curtis and Payne ^32^ discovered that the budget period’s variables and the risk priorities of auditors substantially impact the intention of using CAATs in the UTAUT. The majority of the following researches were conducted in technologically advanced states, whose assessment set-up might be distinct from the evolving states (Greenstein-Prosch *et al.*, 2008). Such as the US setting, where the audit firms are parted among the Big 4, national, local, and regional firms. Whereas in Malaysia, apart from the Big-4 and middle-sized regional and national firms, the majority of the audit firms are individual administrators or contains fewer than five local-based associates. ^75^ Hence, the audit firms of Malaysia are likely to be partitioned into the ones focusing on the national or local extent.

Besides, the former studies have limitations since just the technologically featuring aspects were determined there, leaving the atmospheric and organizational impacts untouched. The Technology-Organization-Environment (TOE) context was utilized to remove those limitations, allowing the investigators to scrutinize multiple elements associated with technology, organizations, and the environment. ^76^ Tornatzky, Fleischer ^76^ established the TOE, an acceptance context at the firms-level. The TOE projected the significance of several relevant aspects in adopting novel technologies. The TOE context is largely used in the IT or IS works and is utilized in considering the acceptance characteristics in many IT frameworks. This context is also suitable for studying CAATs acceptance as it ranges over the technological models for involving the standpoints of organizations and the environment. ^77^ However, a query about the range over which the variables might impact CAATs acceptance rises here. Therefore, a CAATs acceptance paradigm was proposed to combine the technical aspects of Diffusion of Innovation (DOI) theory to involve the elements of organization and environment.

The profile of auditees has an impact on auditors’ acceptance of audits. Age, gender, and experience are the elements contained in the profile of an auditee. The term “age” refers to a person’s chronological age as measured in years. A Bachelor’s degree was found to have a direct, significant, and moderate effect on age on user behavior and adoption. ^40^^,^^73^^,^^78^ The sexual classifications of users, both males, and females are referred to as gender. Gender was found to substantially impact technology adoption in businesses in previous research by Venkatesh, Morris. ^40^ Gender moderates the effect of believed benefits on behavioral intention. The number of years that an auditing firm has been in operation is referred to as experience. Previous behavior is influenced by previous experiences. ^72^ Evidence has revealed that prior familiarity or experience with an existing system can assist employees in adjusting to a new similar approach in the workplace. ^79^^,^^80^ Thus, the auditor’s information technology experience is taken into account in this study. Like Bierstaker, Janvrin ^9^ and Mahzan and Lymer, ^29^ we discover proof supporting the thought that working with condition emphatically impacts the inward evaluators’ expectations to utilize and acknowledge CAATs. The outcome recommends that organizations ought to put sufficient cash in cutting edge foundation to relieve the hindrance of inspectors from tolerating and using CAATs. Besides, firms might expand CAATs use however growing new arrangements in regards to recruiting and advancement of examiners. These arrangements ought to devote more weight to inspector’s capacity to utilize information examination in their everyday review exercises.

## Methods

### Ethical approval

This study was approved by Universitas Islam Bandung Ethical Clearance Commite (Protocol Number 718/B.04/Bak-k/XII/2020) after due consultation, consent letter had been provided by the researchers to all respondents. Respondents had provided their consent without any force from anyone. Subsequently, in order to protect the rights and privacy of the respondents, all forms of data were acquired will remain confidential. Written, informed consent was obtained from participants.

### Design and data collection techniques

A quantitative study using a survey method ^94^ has been utilized in this study. Questionnaires were distributed from Juny 2021 to September 2021 to the targeted respondents, BPK personnel, who worked as auditors in the Indonesian public sectors using an online survey. There were 3600 auditors located in the central and regional offices of Indonesia. The criteria used to select the location sites were based on those offices with a significant number of corruption cases and auditees. A total of 400 questionnaires model e-audit were distributed to the targeted respondents. From the 400, 225 surveys were returned and completed. This study combines both studies by Venkatesh, Morris ^40^ on technology adoption and Dowling ^74^ on the use of the system to help do audit works.

The UTAUT model usage from Venkatesh, Morris ^40^ was adopted in this study because it combines several models that predict technology usage, including TAM, ^21^ planned behavior theory, ^72^^,^^81^ the idea of innovation diffusion, ^82^ and the theory of social cognitive learning. ^82^^,^^83^ Furthermore, the UTAUT model has explained 70 percent of the intention variants to use technology and outperform each high-level model. ^40^ The definition for each of the constructs is listed in Table 1. Table 1 is a summary table made by researchers based on several previous studies.

**Table 1.** Variable operation.

Variable	Construct/Indicators
Perfromance expectancy (PE)	CAATs appear to be useful in carrying out my auditing duties
	I was able to complete my audit swiftly thanks to the utilization of CAATs
	The use of CAATs improves the efficiency of my auditing work
	The use of CAATs allows me to complete audits in a short amount of time
	My auditing efficiency improves when I employ CAATs
Effort Expectancy (EE)	The application of CAATs is quite clear and simple to comprehend
	It is simple for me to learn how to use CAATs
	CAATs are simple to use in my opinion
	For me, learning how to use CAATs is simple
Social influence (SI)	People in my immediate environment influence my decision to use CAATs
	My office's most powerful person says that I should use CAATs
	In the use of CAATs, my team's chief auditor is helpful
	Overall, my workplace has been supportive of the use of CAATs
Facilitating condition (FC)	I have the human resources required to use CAATs.
	I have the necessary skills to use CAAT's IT. If I have any problems utilizing CAATs, technicians are always
	I intend to use CAATs in the future, and I will most likely use CAATs in the future.
Intention to use CAATs (BI)	In the future, I intend to use CAATs
	I will most likely use CAATs in the future
	There have been occasions in the previous 3-4 months when I have not used CAATs as effectively as I should have.
Appropriate use of CAATs (AU)	I am now using CAATs in my audit

Source: Venkatesh, Morris ^40^ and Dowling. ^74^

### The respondents

The criteria for respondents in this study were auditors who work at the central and regional offices with a high number of auditees and a high level of corruption cases. BPK’s regional offices or representations include BPK’s Central Java, West Java, and East Java. Regional office with low cases and with a low number of auditees not include the criteria of respondents. The final sample consists of 225 respondents who were the auditors working in the public sector of Indonesia. The distribution of respondents are depicted in Table 2, with the majority of the respondents being men (67.1 percent), aged 36 to 40 (30.2 percent), and having Bachelor’s educational backgrounds (54.7 percent).

**Table 2.** Descriptive statistics on respondents.

Measure	Item	Frequency	(%)
Gender	Male	151	67.1
	Female	74	32.9
Age	26-30	10	4.4
	31-35	70	31.1
	36-40	68	30.2
	41-45	47	20.9
	46 and above	30	13.3
Education	Bachelor	123	54.7
	Magister	101	44.9
	Doctoral	1	0.4
Audit Board Regional Offices	Center/Secretariat General	44	19.6
	East Java Regional	85	37.8
	Central Java Regional	50	22.2
	West Java Regional	46	20.4

Source: Statistical Processing Results, 2022.

### Data analysis

This study has adopted Structural Equation Modelling (SEM) to examine the correlations between factors influencing intention and behavior of using CAATs. Respondents’ profile (gender, age, and experience) has been used as a moderator in this study. The Partial Least Squares (PLS) method was used to analyze the data. The SmartPLS 3 was used to analyze the data for correlations between factors influencing intention and behavior of using CAATs.

## Results

### Reliability and validity tests

The purpose of the reliability and validity tests was to determine whether the construct matched the structural model domain analysis. Cronbach Alpha (CA) was used to measure the reliability test, and the Fornell-Larcker measure was calculated from the composite reliability score (CR). The mean-variance extracted was used to conduct a validity test (AVE). The recommended score of the CA and CR for each construct is above 0.70, ^84^^,^^85^ and the AVE score of all factors should meet the recommended cutting-of point of 0.5 and above. ^86^ Table 3 shows the results of the CA, CR, and AVE tests. The value of CA and CR scores for each construct is more significant than 0.70, and it is within the allowable reliability range. All factors in the AVE satisfy the recommended cutting-off point of 0.5 and higher.

**Table 3.** The results testing of CA, CR, AVE, dan VIF.

Factor	CA	CR	AVE	VIF	
				AU	BI
PE	0.892	0.920	0.698		2.241
EE	0.897	0.928	0.763		2.264
SI	0.830	0.888	0.665		2.310
FC	0.764	0.864	0.680	1.473	
BI	0.872	0.921	0.796	1.453	
AU	1.000	1.000	1.000		
PE_age	1.000	1.000	1.000		2.322
PE_sex	1.000	1.000	1.000		2.048
EE_age	1.000	1.000	1.000		2.394
EE_edu	1.000	1.000	1.000		1.555
EE_exp	1.000	1.000	1.000		7.937
EE_sex	1.000	1.000	1.000		1.736
SI_age	1.000	1.000	1.000		1.743
SI_edu	1.000	1.000	1.000		1.704
SI_exp	1.000	1.000	1.000		8.764
SI_sex	1.000	1.000	1.000		2.055
FC_age	1.000	1.000	1.000	1.397	
FC_edu	1.000	1.000	1.000	1.333	
FC_exp	1.000	1.000	1.000	1.499	
age	1.000	1.000	1.000	1.151	1.305
edu	1.000	1.000	1.000	1.115	1.169
exp	1.000	1.000	1.000	1.473	1.685
sex	1.000	1.000	1.000		1.064

Source: Statistical Processing Results, 2022.

### Multicollinearity test

The variance inflation factor (VIF) was used to test for multicollinearity problems. It is used to identifies the correlation between independent variables and the strength of that correlation. Because the value of VIF for the constructs is less than the maximum cutting-off point of 10, the structural study model is not negatively affected by the issue of collinearity. ^87^ The results are depicted in Table 3. As the results indicates, the value of variance inflation factor for all varibales is higher than 1, which demonstrates the moderate correlation between variables.

### Discriminant validity

**Figure 1.**
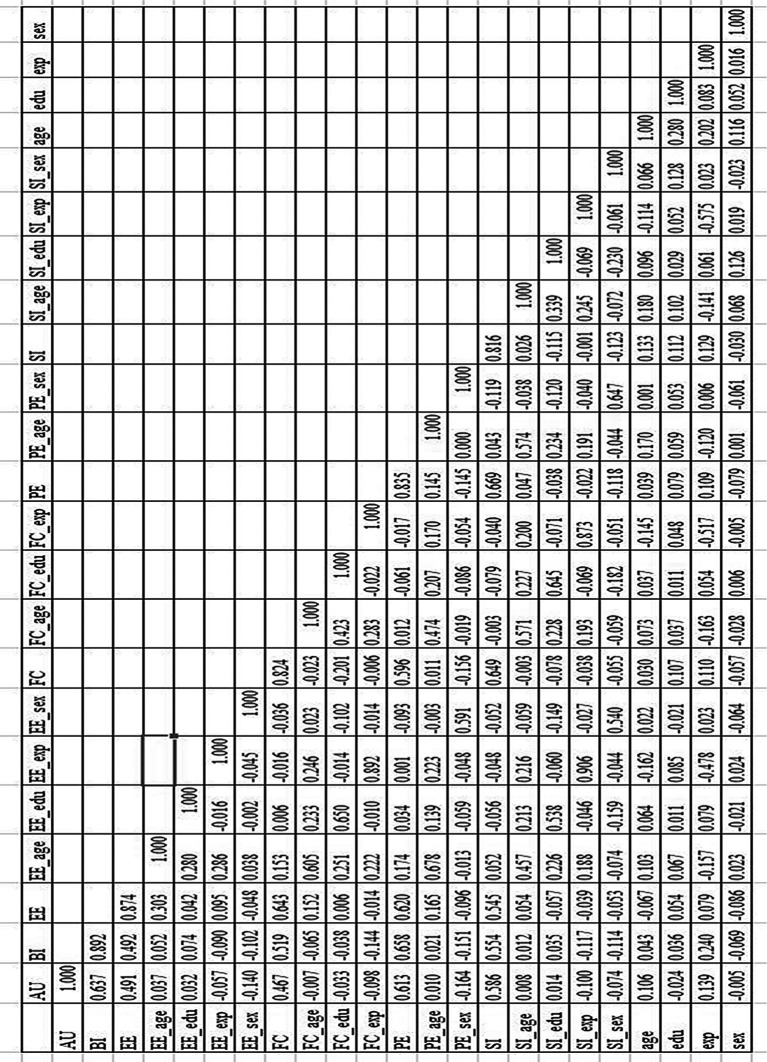
The results of discriminant validity test.

The square root of the AVE score is used to determine the validity of this model. A factor must be higher than the factors’ cross-correlation. ^84^ Figure 1 shows the results of the discriminant validity test, which shows that discriminant validity is reliable because the AVE scores for those factors are higher than the square cross-correlation for those factors.

### Structural model analysis

**Figure 2.**
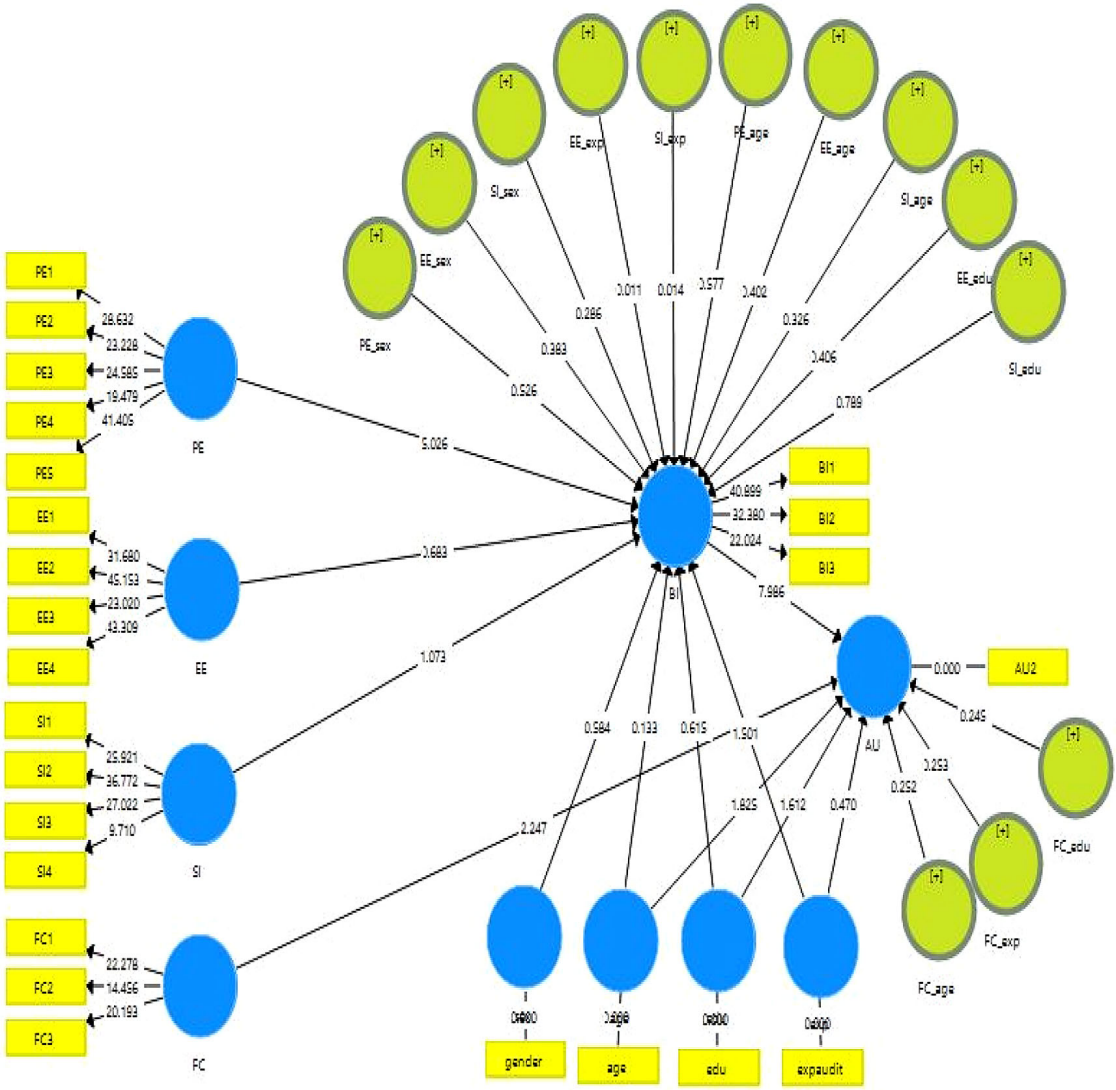
Research structural model. (Source: the results of the author’s analysis using the SmartPLS software).

The structural model was carried out after testing the constructs for reliability test, validity, discriminant, and multicollinearity tests. Next, the hypotheses were tested using the Bootstrap Smart-PLS technique. As shown in Figure 2 and Table 4, the structural model results indicate the correlation between exogenous and endogenous factors using an algorithm of PLS.

**Table 4.** Regression result.

Hypothesis	β	STDEV	T Statistics	p-value	Result
PE -> BI	0.464	0.092	5.052	0.000	Accepted
EE -> BI	0.116	0.175	0.664	0.421	Rejected
SI -> BI	0.175	0.159	1.106	0.269	Rejected
FC -> AU	0.202	0.091	2.226	0.027	Accepted
BI -> AU	0.538	0.073	7.341	0.000	Accepted
PE_age -> BI	-0.061	0.098	0.623	0.533	Rejected
PE_sex -> BI	-0.048	0.098	0.490	0.624	Rejected
EE_age -> BI	-0.041	0.102	0.400	0.689	Rejected
EE_edu -> BI	0.029	0.073	0.402	0.688	Rejected
EE_exp -> BI	-0.008	0.645	0.012	0.991	Rejected
EE_sex -> BI	-0.027	0.067	0.408	0.684	Rejected
SI_age -> BI	0.031	0.093	0.331	0.741	Rejected
SI_edu -> BI	0.073	0.090	0.813	0.417	Rejected
SI_exp -> BI	0.008	0.554	0.015	0.988	Rejected
SI_sex -> BI	0.032	0.120	0.268	0.789	Rejected
FC_age -> AU	0.023	0.081	0.285	0.776	Rejected
FC_edu -> AU	0.017	0.072	0.241	0.810	Rejected
FC_exp -> AU	-0.022	0.064	0.349	0.727	Rejected
R2 AU	0.447				
R2 BI	0.507				
Q2 AU	0.377				
Q2 BI	0.353				

Gender, education, and audit experience do not play a role in moderating the links between effort expectancy, performance expectancy, and social influence in facilitating behavior intention and usage intention (p>0.05). Interestingly, there is a favorable association between the intention of using CAATs and using CAATs appropriately. ^93^

Performance expectancy is positively related to the intention to use CAATs, and facilitating conditions are positively associated with the appropriate use of CAATs. The coefficient determinant (R ^2^) is used to assess the model quality test. ^86^ The estimated R ^2^ for correctly using CAATs is 0.447 (44.7 percent), indicating that exogenous factors are 0.447 (44.7 percent) in the endogenous variables of appropriately using CAATs. Meanwhile, the calculated R2 for the intention of using CAATs is 0.507 (50.7 percent), indicating that exogenous factors are 0.507 (50.7 percent) in the endogenous variables of “Intention to use CAATs.” A good model is where the fit model test employs cross-validated redundancy (Q2), more significant than zero. ^86^^,^^88^ In sum, this model is fit or has a predictive interest based on the results of Q2AU, which is 0.377, and Q2BI, which is 0.353 ( Table 4).

## Discussion

Performance expectancy has a positive and significant influence on the intention to use CAATs. Thus H _1_ is accepted. Auditors believe that employing CAATs in auditing will help them execute tasks more efficiently. This also indicates that BPK’s auditors believe and agree that CAATs help assists their auditing works. The positive association between performance expectations and the intention to use CAATs has also been observed in previous studies. ^9^^,^^29^^,^^50^^,^^68^

However, the results of different tests for the variable constructs of effort expectancy (H _2_) on the use of CAATs indicate no statistically significant association. This finding differs from past studies. ^1^^,^^68^ The existence of four CAATs applications given by BPK still requires more intense training to the staff, which may explain why effort expectancy does not stimulate the intention to utilize CAATs. Another possibility is that evaluated entities (auditees) are considered sufficient to audit without CAATs, which is more likely to occur. However, these are not found in this study, implying that more research and exploration are required to uncover relevant essential elements. The finding reveals that effort expectancy is unrelated to using CAATs is consistent with previous research by Mahzan and Lymer. ^29^

The significance of the job of CAATs in the review interaction is generally perceived. However, notwithstanding their normal advantages and ideas from researchers and controllers, various inward reviewers do not right now embrace these instruments when directing different inward review capacities. ^34^ Acquiring from data frameworks research, we utilize the UTAUT model to analyze the determinants of CAATs reception by inward examiners in Jordan. After reviewing 105 inward examiners, this investigation discovers that the significant variables which might influence inside inspectors’ goals to utilize CAATs are working with conditions and execution anticipation. Be that as it may, both exertion hope and social impact are observed to be inconsequential.

As discussed before, firms can grow CAATs utilization by instructing inside evaluators concerning the advantages of utilizing these automated devices, by devoting more response to put resources into the specialized foundation, by working on their abilities through expanded CAATs preparing programs, and by creating reward frameworks that urge reviewers to utilize CAATs. Second, considering that the choice to acknowledge and use CAATs is willful, firms ought to perceive the results of their strategies and culture on inner reviewers’ aim to take on CAATs. ^34^ In conclusion, our review proposes the putting resources into review programming without thinking about the hindrances to the reception of CAATs, may restrict the ideal impacts of these robotized devices.

The result of variable construct testing of social influence on the intention of CAATs usage (H _3_) is not significant, consistent with previous studies. ^9^^,^^29^^,^^67^ In BPK, social influence on the intention of CAATs usage is insignificant because their location is far from each entity, and they have low access to an internet connection. According to the Speedtest Global Index, Indonesia is among the Asia Pacific countries with a standard internet connection. ^89^

In particular, the relapse investigations show that the impact of execution hope, as predicted by H _1_, was genuinely huge in clarifying the expectation of internal auditors to adopt on CAATs. This outcome is predictable. ^9^^,^^29^^,^^32^ This outcome recommends that internal evaluators be more ready to use CAATs when they know that the advantages acquired from utilizing these mechanized apparatuses would further develop their work productivity. Accordingly, the board that looks to expand CAATs utilization ought to prepare projects to instruct inward reviewers about the advantages of using such devices and assist them with staying current with evolving innovation. ^9^

The variable construct of the facilitating condition (H _4_) was found to be significant and has a positive influence on the appropriate use of CAATs. ^1^^,^^9^^,^^29^^,^^50^^,^^68^ Additionally, the construct of the intention of using audit (H _5_) has a positive association with the appropriate use of CAATs. ^74^ This implies that the facility of information technology is available to support the proper use of CAATs.

As per past research, ^29^^,^^32^ we find that work expectancy is irrelevant. The reasons behind the irrelevant outcome might be that the more significant part of inward inspectors in our example are youthful and have an undeniable degree of capability in data innovation. Hence, interior evaluators might see that the level of straightforwardness with the utilization of CAATs is generally irrelevant to their choice. One more translation for the irrelevant outcome is that in an examining setting, the viability of review strategies is given a high need by inside evaluators when settling on innovation use choices instead of the individual inclinations concerning the endeavors needed to utilize the innovation. ^9^ Despite our assumption, the discovering shows that social impact is inconsequential, implying that that choice to utilize CAATs is not influenced by the prevailing difficulty emerging from the head of the inside review division, their friends inside the organizations, or from the expert bookkeeping bodies. In this specific situation, Venkatesh, Morris ^40^ state that the social impact factor is huge in an acute setting, though it is not huge in an intentional setting. In Jordan, CAAT’s use is willful, despite various researchers and expert guidelines urging reviewers to utilize CAATs.

What is more, Mahzan and Lymer ^29^ noticed that the level of requirement and checking of consistency with CAATs utilization is powerless, thus making internal auditors allowed to perform setting explicit choices on reception. Therefore, the willfulness of utilization has no impact on the goal to utilize CAATs. Generally speaking, the proof is conflicting with Curtis and Payne ^32^ and Gonzalez, Sharma, ^90^ yet is in accordance with previous studies. ^9^^,^^29^^,^^32^

Also, multiple studies concentrate significantly on computer-based audit support systems. Thompson, Higgins ^71^ recognized the technology’s influence on audit preparation in five big audit firms and stated that technology might offer consumer-specific interior controls that help the auditor identify the customer’s system lacking. Additionally, it was also revealed that technology helps to examine the business procedures of clients, for considering and measuring the degree of control, as well as suggests trials being required to conduct. Moreover, the technology assures concurrence with audit standards and further guidelines associated with the audit. Bierstaker, Burnaby ^51^ explained the technology used in the audit procedure by interviewing IT experts from four of the five biggest accounting firms in the US. The studies of Bierstaker, Burnaby ^51^ were descriptive by nature and concentrated on an individual audit application from an audit firm; therefore, both studies cannot be generalized to the exterior auditor’s proper technology usage. ^91^

Like Bierstaker, Janvrin ^9^ and Mahzan and Lymer, ^29^ we discover proof supporting the thought that working with conditions definitely impacts the inward evaluators’ expectations to utilize and acknowledge CAATs. The outcome recommends that organizations ought to put sufficient cash in a cutting-edge foundation to relieve the hindrance of inspectors from tolerating and using CAATs. Besides, firms might expand CAATs use, however, growing new arrangements regarding recruiting and advancement of examiners. These arrangements ought to devote more weight to inspector’s capacity to utilize information examination in their everyday review exercises.

Both scholars and practitioners will benefit from these discoveries. The audit profession is “developing rapidly as a result of innovation in its environment,” according to Solomon and Trotman. ^4^ As a result, the audit profession is under pressure to increase audit efficiency and effectiveness. ^92^ Because both researchers and practitioners contend that CAATs will improve audit efficiency and effectiveness, our findings may aid both researchers and practitioners in their efforts to boost CAATs acceptability. Furthermore, previous research only looked at a small number of CAATs with small sample sizes and an emphasis on the prevalence of CAATs rather than the underlying reasons for their usage. ^41^

In contrast, our study aims to examine public sector auditors’ tendency to use Computer Assisted Audit Techniques (CAATs). This study covers broad areas of Central Java, West Java, and East Java. Further, the findings add to the literature on emerging markets specifically for Indonesian government auditors’ intention and appropriateness of using CAATs. The use of CAATs help to provide auditors information on the highest number of auditees involved in corruption. The empirical findings reveal that performance expectation and facilitating conditions have encouraged auditors to use CAATs in their works. Further, there is a positive influence between the intention to use and CAATs audit. This implies that auditors with an intention will be more open to use the CAATs optimally in achieving effective and efficient work. The utilization of CAATs in public services need to have strong support from the government and positive attitudes from the auditors as the users of the system.

## Conclusion

The use of CAATs in today’s audits has become a critical issue in achieving efficiency and effectiveness. Therefore, this study examines the use of CAATs by auditors in the public sector of Indonesia. The UTAUT model measures the performance expectancy, effort expectancy, social influence, facilitating conditions, the intention of CAAT usage, and the appropriate use of CAATs. Findings evidence that the performance expectations and the facilitating conditions have shown a positive association with the use of CAATs in doing audits. Further, the result reveals that a positive influence between the intention to use and CAATs audit. This shows that an individual with a strong intention will be more inclined and excited to utilize the CAATs to be used appropriately and optimally based on its goal. The readiness of the CAATs system and the support of both facilitating conditions will increase an auditor’s desire to use CAATs. Furthermore, a detailed and extensive audit will improve performance expectancy by examining the role of CAATs. It can improve audit performance by being faster, more effective, and efficient.

There are some limitations identified in this study. The CAATs in the BPK are still new and are undergoing the process; thus, the impact may not be stable and cannot be justified at large. Next, no in-depth interview is conducted to gain a better insight into the CAATs employed at BPK. Supposedly, the interview can provide a better view of the respondents’ perceptions on the effectiveness of CAATs implementation. Thus, the future study may consider all these factors to be researched in detail.

## Data availability

### Underlying data

Figshare: Dataset of Questionnaire Result from the respondents of Modelling Computer Assisted Audit Techniques (CAATs) in Enhancing Indonesian Public Sector, https://doi.org/10.6084/m9.figshare.19642374.v2. ^93^

This project contains the following underlying data:

- Response from 225 respondents in Model E-audit Indonesia.csv

### Extended data

Figshare: List of of questions and descriptions of questionnaire of the Modelling Computer Assisted Audit Techniques (CAATs) in Enhancing Indonesian Public Sector, https://doi.org/10.6084/m9.figshare.19642437.v1. ^94^

This project contains the following extended data:

- List of of questions.csv

Figshare: The Respondent characteristics of the Modelling Computer Assisted Audit Techniques (CAATs) in Enhancing Indonesian Public Sector, https://doi.org/10.6084/m9.figshare.19642425.v2. ^95^

This project contains the following extended data:

- The Respondent characteristics.csv

Data are available under the terms of the Creative Commons Attribution 4.0 International license (CC-BY 4.0).
