# Supplementary material for: Modelling computer assisted audit techniques (CAATs) in enhancing the Indonesian public sector
Source: F1000Res. 2022 Oct 31;11:559. Originally published 2022 May 23. [Version 2] doi: 10.12688/f1000research.121674.2 (PMC9699992; doi:10.12688/f1000research.121674.2)
Supplement: Peer review history

# Peer review: Reviewer response for version 2

**DOI:** 10.5256/f1000research.139894.r154538

**Published:** 2022-11-25

**Authors:** Ellis Kofi Akwaa-Sekyi

**Affiliations:** 1 Kwame Nkrumah University of Science and Technology, Kumasi, Ghana

The authors have incorporated most of the suggestions and comments made in the initial edition. However, these observations are worth considering:

 The authors must be specific with the exact contribution of the study. Show in what ways specific findings contribute to research. What you have written is a generic statement.

 There are too many redundant references. You may consider reducing the references to 50. It has unnecessarily bloated the word count.

 The work still requires proofreading

Is the work clearly and accurately presented and does it cite the current literature?

Partly

If applicable, is the statistical analysis and its interpretation appropriate?

Yes

Are all the source data underlying the results available to ensure full reproducibility?

Yes

Is the study design appropriate and is the work technically sound?

Yes

Are the conclusions drawn adequately supported by the results?

Partly

Are sufficient details of methods and analysis provided to allow replication by others?

Partly

Reviewer Expertise:

Corporate governance, Internal controls, Venture capital finance, Bank risk

I confirm that I have read this submission and believe that I have an appropriate level of expertise to confirm that it is of an acceptable scientific standard.

# Peer review: Reviewer response for version 1

**DOI:** 10.5256/f1000research.133566.r147101

**Published:** 2022-09-28

**Authors:** Ellis Kofi Akwaa-Sekyi

**Affiliations:** 1 Kwame Nkrumah University of Science and Technology, Kumasi, Ghana

The paper examines how computer assisted audit techniques enhances the auditor’s work in the Indonesian public sector. This is a trending subject in modern day auditing and financial reporting. The introduction of ICT into the auditing profession calls for studies of this nature to unearth the intricacies involved in this transformation agenda. In spite of the effort exerted in the writing of this manuscript, it is believed that if the authors pay attention to these suggestions, the quality of the paper will improve tremendously:

- Rewrite the conclusion section of the abstract. It is only the last sentence which looks like a conclusion.
- The introduction is too long. It should specifically cover the purpose of study, research gaps, problem statement, motivation for the study, brief methodology and results (optional), the contribution of the study and structure of the paper. What is it that is missing in research that is necessitating this paper?
- The authors should use the literature review section to amplify the research gaps. Show from the literature review that there is still a missing link in spite of the plentiful research on the topic. This will strengthen the significance of the study. Otherwise, an impression is created that that there is nothing more to research about.
- The voice of the literature review section should not sound like a recommendation.
- Why do you have only one question on the actual use of CAATs? It gives the impression that the study is focused on intention more than behavior.
- The authors fail to show a scientific approach to the determination and selection of the respondents.
- The discussion component should not be mixed with the limitations, implications and conclusions. It is good practice to use the discussion to relate the findings to existing literature and proposed underlying theories. The authors should strengthen why most of their hypotheses were rejected. Is that not a cause for concern?
- The authors should use the conclusion section to answer the research questions. After a brief summary of the work, the key research questions should be answered. How were the limitations addressed such that they did not affect the results of the study?
- The work heavily relies on literature that is a bit old. Even though the topic is a current issue, a significant number of the references are old. The authors may consider beefing up the references with current ones.
- The manuscript requires thorough proofreading and editing.

Is the work clearly and accurately presented and does it cite the current literature?

Partly

If applicable, is the statistical analysis and its interpretation appropriate?

Yes

Are all the source data underlying the results available to ensure full reproducibility?

Yes

Is the study design appropriate and is the work technically sound?

Yes

Are the conclusions drawn adequately supported by the results?

Partly

Are sufficient details of methods and analysis provided to allow replication by others?

Partly

Reviewer Expertise:

Corporate governance, Internal controls, Venture capital finance, Bank risk

I confirm that I have read this submission and believe that I have an appropriate level of expertise to confirm that it is of an acceptable scientific standard, however I have significant reservations, as outlined above.

# Response

**Published:** 2022-10-15

**Authors:** Pupung Purnamasari

**Affiliations:** Universitas Islam Bandung, Indonesia

Dear reviewers,

 We have made revisions according to your suggestions.

# Peer review: Reviewer response for version 1

**DOI:** 10.5256/f1000research.133566.r145799

**Published:** 2022-08-02

**Authors:** Agung Nur Probohudono

**Affiliations:** 1 Department of Accounting, Universitas Sebelas Maret Surakarta, Surakarta, Indonesia

I have read this paper and I believe it deserves to be published. This is intended to provide further information to all parties related to reporting in the public sector to improve their performance in terms of strengthening audits.

 The problems that arise are in accordance with the real conditions and the given background is logical. The author has described that many organizations have used information technology to develop and improve the effectiveness of their performance. Utilization of information technology is also an effective means of improving the quality of public services. However, its utilization in Indonesia is still minimal, so it needs encouragement from various parties, especially from the government.

 In the future, it is necessary to provide data on how much or the current level of usage in Indonesia.

 The method offered is good although it can still be enforced by sensitivity analysis. Respondents have covered the minimum needs of data analysis and are able to represent the desired conditions in this study.

 The sensitivity analysis we mean is to be able to find out more about the difference between the results obtained with the analysis carried out by analysis with other methods, whether it will show the same results.

 The results of the analysis are presented completely and clearly, and show that there are still many factors that have no effect. This is the reason that this research is important to be developed again in the future so that the implementation of CAATs can be optimized.

 The conclusions that have been drawn are good for reference by various interested parties, where the use of CAATs in today's audits has become an important issue in achieving efficiency and effectiveness, especially in the public sector. The conclusions in this study have also provided a clear direction for future research.

Is the work clearly and accurately presented and does it cite the current literature?

Yes

If applicable, is the statistical analysis and its interpretation appropriate?

Yes

Are all the source data underlying the results available to ensure full reproducibility?

Partly

Is the study design appropriate and is the work technically sound?

Yes

Are the conclusions drawn adequately supported by the results?

Yes

Are sufficient details of methods and analysis provided to allow replication by others?

Yes

Reviewer Expertise:

Public sector, Financial reporting, Financial statement analysis, Internal control, Corporate governance

I confirm that I have read this submission and believe that I have an appropriate level of expertise to confirm that it is of an acceptable scientific standard.
